# Identification of New Molecular Biomarkers in Ovarian Cancer Using the Gene Expression Profile

**DOI:** 10.3390/jcm11133888

**Published:** 2022-07-04

**Authors:** Piotr Józef Olbromski, Piotr Pawlik, Anna Bogacz, Stefan Sajdak

**Affiliations:** 1 Clinic of Operational Gynecology, Poznan University of Medical Sciences, Polna 33, 60-535 Poznan, Poland; olbromski.piotr@gmail.com (P.J.O.); pitpawlik@gmail.com (P.P.); kgo.sekretariat@gpsk.ump.edu.pl (S.S.); 2 Department of Stem Cells and Regenerative Medicine, Institute of Natural Fibers and Medicinal Plants, Kolejowa 2, 62-064 Plewiska, Poland

**Keywords:** ovarian cancer, gene expression, biomarkers, protein expression

## Abstract

Ovarian cancer is a common cause of death among women worldwide. The current diagnostic and prognostic procedures available for the treatment of ovarian cancer are either not specific or are very expensive. Gene expression profiling has proved to be a very effective tool in the exploration of new molecular markers in patients with ovarian cancer, although the link between such markers and patient survival and clinical outcomes is still elusive. We are looking for genes that may function in the development and progression of ovarian cancer. The aim of our study was to evaluate the expression of selected suppressor genes (ATM, BRCA1, BRCA2), proto-oncogenes (KRAS, c-JUN, c-FOS), pro-apoptotic genes (NOXA, PUMA), genes related to chromatin remodeling (MEN1), and genes related to carcinogenesis (NOD2, CHEK2, EGFR). Tissue samples from 30 normal ovaries and 60 ovarian carcinoma tumors were provided for analysis of the gene and protein expression. Gene expression analysis was performed using the real-time PCR method. The protein concentrations from tissue homogenates were determined using the ELISA technique according to the manufacturers’ protocols. An increase in the expression level of mRNA and protein in women with ovarian cancer was observed for KRAS, c-FOS, PUMA, and EGFR. No significant changes in the transcriptional levels we observed for BRCA1, BRCA2, NOD2, or CHEK2. In conclusion, we suggest that KRAS, NOXA, PUMA, c-FOS, and c-JUN may be associated with poor prognosis in ovarian cancer.

## 1. Introduction

Ovarian cancer causes the highest number of deaths of any gynecological cancer. The disease is initially asymptomatic, and therefore, it is usually detected in advanced stages, when a complete cure is almost impossible [1]. In 2018, approximately 293,000 new cases of ovarian cancer were diagnosed, and 185,000 cases resulted in death [2]. It is estimated that 65% of all ovarian cancers are diagnosed as advanced-stage (FIGO III–IV), leading to a 5-year overall survival (OS) of patients ranging from 30 to 50% [3]. Despite many years of research, there are still no biomarkers with appropriate diagnostic sensitivity and specificity or other effective diagnostic methods enabling screening. The pathogenesis of ovarian cancer is still unclear; the suspected etiology indicates the involvement of hormones and genetic and environmental factors [4,5,6].

A characteristic feature of malignant tumors and the main cause of death from cancer is metastasis. In the multistage process of neoplasm, the accumulation of mutations and changes in gene expression occurs, which is associated with the acquisition of not only the characteristics of a growing phenotype but also an invasive phenotype, characteristic of a malignant tumor, with the ability to overcome the basal membranes of vessels, tissues, and organs and to initiate secondary growth in places distant from the primary lesion. Despite many attempts, molecular tests in the early diagnosis of ovarian cancer have not yet been established [7,8,9,10]. Analysis of the expression profile of genes associated with the tumorigenesis process is an important research strategy that combines data from genetics and molecular transcription to find dysregulated genes between patients and healthy individuals [7]. Microarray research is providing more and more whole-gene transcription information for ovarian cancer. Moreno et al. showed that cell proliferation, apoptosis, the cell cycle, and DNA damage were upregulated in ovarian cancer [8]. Another study showed changes in the expression levels of genes acting in several signaling pathways, such as Wnt, Notch, TGFβ/BMP, and the canonical cell cycle [9]. Oliveira et al. found four candidates—HSPA1A, CD99, RAB3A, and POM121L9P, which are associated with overall survival and poor clinicopathological features [10]. However, the results of the previous studies differ due to the diversity of the selection of the cohort, the origin of the samples, and experimental designs. Therefore, we are constantly looking for genes that may function in the development and progression of ovarian cancer. The aim of our study was to evaluate the expression of selected suppressor genes (ATM, BRCA1, BRCA2), proto-oncogenes (KRAS, c-JUN, c-FOS), pro-apoptotic genes (NOXA, PUMA), genes related to chromatin remodeling (MEN1), and genes related to carcinogenesis (NOD2, CHEK2, EGFR).

## 2. Materials and Methods

### 2.1. Patients

A total of 90 subjects were recruited for the study, including 60 patients with ovarian cancer diagnosed and treated at the Clinical Hospital of the Poznan University of Medical Sciences (Poland). Histological tests were the basis of cancer diagnosis. The majority of patients in the study group had serum ovarian cancer, stage III according to FIGO (International Federation of Gynaecology and Obstetrics), with a low degree of differentiation. The mean age of these patients was 57 years (41 to 80 years).The control group (n = 30) included women operated on for uterine fibroids or prolapse of the reproductive organ after menopause without any history of cancer. The mean age of these patients was 63 years (51–77 years). Tissue samples from 30 normal ovaries and 60 ovarian carcinoma tumors were provided for analysis of gene and protein expression.

The Bioethics Committee of Poznan University of Medical Sciences, Poland (no. 77/19), approved the study. All patients were informed about the purpose of the study and provided written informed consent. The study was conducted in accordance with the Declaration of Helsinki.

### 2.2. Expression Analysis

The RNA isolation from tumor tissue removed during surgery was performed using TriPure Isolation Reagent (Roche Diagnostics, Mannheim, Germany), according to the manufacturer’s protocol. cDNA synthesis from total RNA was performed using the Transcriptor First Strand Synthesis Kit (Roche Diagnostics, Mannheim, Germany). The obtained cDNA was used directly for the real-time PCR (RT-PCR) or stored at −20 °C. The mRNA levels of genes such as ATM, BRCA1, BRCA2, KRAS, c-JUN, c-FOS, NOXA, PUMA, MEN1, NOD2, CHEK2, and EGFR were analyzed by real-time quantitative PCR using a LightCycler480 Instrument (Roche, Mannheim, Germany) and a LightCycler480 Probes Master kit (Roche, Mannheim, Germany). GAPDH and β-ACTIN were used as housekeeping genes for normalization. All primer sequences were synthesized by Genomed (Warsaw, Poland) and are summarized in Table 1. The PCR program was initiated with activation at 95 °C for 10 min. Each PCR cycle comprised a denaturation step at 95 °C, an annealing step at a specific temperature, and an extension step at 72 °C. The increase in the fluorescence level of PCR products was measured, and the data were analyzed using the LightCycler480 software.

### 2.3. ELISA

The Human ATM ELISA Kit (sensitivity: 0.094 ng/mL; LSBio, Seattle, WA, USA), Human BRCA1 ELISA Kit (sensitivity: 0.065 ng/mL; LSBio), BRCA2 ELISA Kit (sensitivity: 0.062 ng/mL; Biomatik, Wilmington, NC, USA), Human KRAS ELISA Kit (sensitivity: 0.115 ng/mL; LSBio, Seattle, WA, USA), C-JUN ELISA Kit (sensitivity: 1.0 ng/mL; MyBioSource, San Diego, CA, USA), Human c-FOS ELISA Kit (sensitivity: 0.188 ng/mL; AssayGenie, Dublin, Ireland), Human NOXA ELISA Kit (sensitivity: 0.078 ng/mL; LSBio, Seattle, WA, USA), Human PUMA ELISA Kit (sensitivity: 0.056 ng/mL; LSBio, Seattle, WA, USA), Human MEN1 ELISA Kit (sensitivity: 0.039 ng/mL; MyBioSource, San Diego, California, USA), Human NOD2 ELISA Kit (sensitivity: 0.062 ng/mL; MyBioSource, San Diego, CA, USA), Human CHEK2 ELISA Kit (sensitivity: 0.062 ng/mL; MyBioSource, San Diego, CA, USA), and Human EGFR ELISA Kit (sensitivity: 0.001 ng/mL; MyBioSource, San Diego, CA, USA) were employed to evaluate the concentrations of ATM, BRCA1, BRCA2, KRAS, c-JUN, c-FOS, NOXA, PUMA, MEN1, NOD2, CHEK2, and EGFR from tissue homogenates, according to the manufacturers’ protocols. The reaction was blocked, and the absorbance was measured on a microplate reader (Infinite 200, TECAN). The concentrations of ATM, BRCA1, BRCA2, KRAS, c-JUN, c-FOS, NOXA, PUMA, MEN1, NOD2, CHEK2, and EGFR were determined by interpolation of the standard curve using linear regression analysis.

### 2.4. Statistical Analysis

Statistical analysis was performed by the SPSS 17.0 PL program using one-way analysis of variance (ANOVA). All values are expressed as means ± SD. Values of *p*< 0.05 were considered statistically significant differences [11].

All methods were carried out in accordance with relevant guidelines and regulations.

## 3. Results

In Table 2, we show the statistical differences in the level of leukocytes, platelets, hemoglobin, hematocrit, glucose, and sodium between the control group and women with ovarian cancer. The values for D-dimer and fibrinogen were statistically higher in women with ovarian cancer compared with controls (3329.63 ng/ML vs.789.69 ng/mL, *p* < 0.001; 7.55g/L vs. 2.91g/L, *p* < 0.001, respectively). The tumor markers CA-125 and HE4 were also statistically higher in patients with ovarian cancer (773.51–454.22 U/mL vs. 128.29 U/mL, *p* < 0.001;1708.42 pmol/L vs. 83.47 pmol/L, *p* = 0.009, respectively).

**Figure 1:**
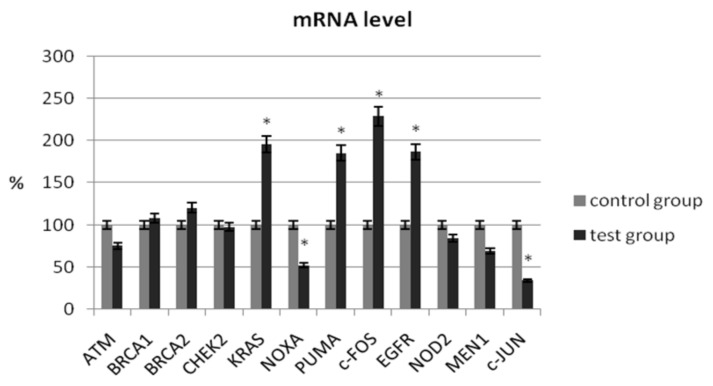
Analysis of mRNA expression of selected genes in patients with ovarian cancer compared with the control group. The control group was defined as 100%. Data are presented as mean% ± SD, * *p* < 0.05 compared with the control group.

In our study, we determined the expression of selected suppressor genes (ATM, BRCA1, BRCA2), proto-oncogenes (KRAS, c-JUN, c-FOS), pro-apoptotic genes (NOXA, PUMA), genes related to chromatin remodeling (MEN1), and genes related to carcinogenesis (NOD2, CHEK2, EGFR) using the real-time PCR method. The analysis of the mRNA level of the ATM gene did not show a significant change in its expression in patients with ovarian cancer compared with the control group (75.45% ± 5.56 vs. 100% ± 4.26; *p* > 0.05) (Table 3). As shown in Figure 1, no significant changes in transcriptional levels were observed for BRCA1, BRCA2, NOD2, or CHEK2. In patients with ovarian cancer, the mRNA level of MEN1 and ATM1 was reduced by 31% (*p* > 0.05) and 25% (*p* > 0.05), respectively.

A significant decrease in mRNA expression level was observed for *NOXA* (51.96% ± 7.34 vs. 100% ± 8.32; *p =* 0.024) and *c-JUN* (34.14% ± 6.42 vs. 100% ± 9.44; *p =* 0.001). A statistical increase in the expression level of selected genes in women with ovarian cancer was observed for *KRAS* (195.55% ± 18.32 vs. 100% ± 15.24; *p =* 0.012), c-FOS (228.57% ± 21.44 vs. 100% ± 12.36; *p =* 0.008), PUMA (184.97% ± 18.42 vs. 100% ± 14.82; *p =* 0.026), and EGFR (186.66% ± 17.54 vs. 100% ± 10.35; *p =* 0.006) (Table 3).

**Figure 2:**
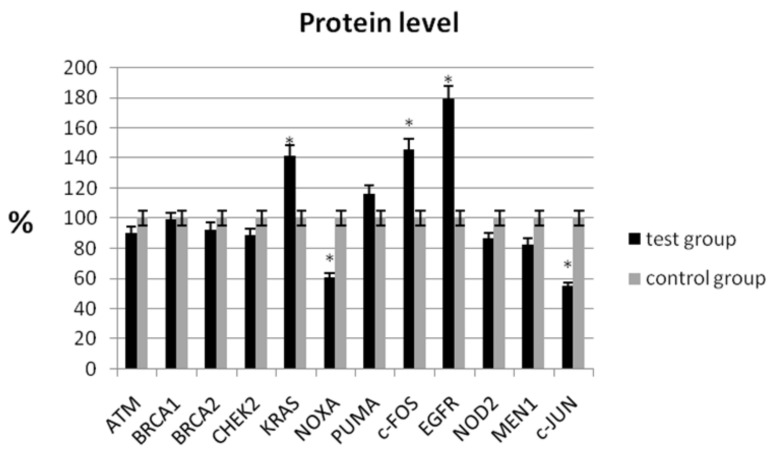
Analysis of protein level of selected genes in patients with ovarian cancer compared with the control group. The control group was defined as 100%. Data are presented as mean% ± SD, * *p* < 0.05 compared with the control group.

In the study, the analysis of the protein level showed a statistically significant decrease in expression for NOXA (60.62% ± 6.56 vs. 100% ± 7.32; *p* < 0.05) and c-JUN (54.43% ± 9.82 vs. 100% ± 8.44; *p* < 0.05) compared with the control group (Table 4, Figure 2). Similarly, a decrease in mRNA expression was observed for these genes (Table 3). We observed an increase in protein levels for KRAS (141.11% ± 8.65 vs. 100% ± 9.68; *p* < 0.05), c-FOS (145.52% ± 10.48 vs. 100% ± 9.46; *p* < 0.05), and EGFR (178.89% ± 7.32 vs. 100% ± 8.98; *p* < 0.05), respectively (Table 4).

## 4. Discussion

Taking into account the current data on ovarian cancer, it appears that approximately 65% of all cases are diagnosed at an advanced stage [3]. It is believed that new biomarkers may be beneficial in improving patient prognosis. Therefore, the discovery of new molecular targets remains a significant clinical challenge in treatment decision-making. In recent years, mRNA assessment has been widely used to identify and develop new molecular biomarkers for the diagnosis and treatment of many types of cancer [19]. Such biomarkers can offer early and more accurate prediction and prognosis of the disease and its progression, allowing the identification of those potentially at risk. Moreover, the assessment of the patient’s mRNA profile additionally enables the identification of not only unique biomarkers but also the association between them [10].

Our study identified genes using real-time PCR and ELISA that are expressed differently in ovarian cancer tissue samples compared to normal ovarian tissue samples. The analysis of the mRNA and protein levels of the ATM and MEN1 showed no change in the expression level in patients with ovarian cancer compared with the control group. An increase in the expression level of mRNA and protein in women with ovarian cancer was observed for KRAS, c-FOS, and EGFR. No significant changes in the transcriptional levels we observed for BRCA1, BRCA2, NOD2, and CHEK2.

Our study design has several advantages in identifying potential ovarian cancer markers over many previous ovarian cancer gene expression studies because a relatively large number of ovarian tissues (30 normal ovaries and 60 ovarian tumors) were used to analyze mRNA and protein expression. By analyzing a large number of tissues, a more accurate expression profile of ovarian cancer genes can be obtained [20,21,22].

Tumor suppressor genes, such as BRCA1 and BRCA2, are involved in inhibiting cell growth and apoptosis, regulating gene transcription, and repairing DNA damage. BRCA mutations are believed to be associated with the risk of developing cancers, especially ovarian and breast cancer [23,24]. Studies conducted by Tsibulak showed higher BRCA1 and BRCA2 mRNA expression in ovarian cancer tissues compared with non-cancerous tissue. The authors explained that the increase in the level of transcription of these genes may be associated with a higher rate of proliferation in malignant tissues and with the process of genetic instability, which may increase the need to repair DNA damage [25]. This suggestion was supported by Gudas et al., who suggested that steroid hormones upregulating BRCA1 expression may be due to increased cell proliferation in breast cancer [26]. Our study did not confirm the results obtained by Tsibulak et al.; however, a slight increase in expression could be observed for the BRCA2 gene. Such a relationship has not been confirmed at the protein level. Another study found that breast cancers with low levels of BRCA2 mRNA expression showed a significantly higher 5-year survival rate [27]. Moreover, Tsibulak et al. showed that in breast cancer with the BRCA1 mutation, mRNA expression for the BRCA1 gene was lower, while BRCA2 expression was significantly higher compared with wild-type BRCA1 cancer [25]. The reason for the “compensatory” upregulation of BRCA2mRNA in tumors with low BRCA1 expression is still speculated, as there is no precise knowledge about the regulation of BRCA protein expression in normal or malignant tissues. The authors explain that high BRCA expression may define a distinct phenotype with high constitutive expression or may reflect transient upregulation induced by various situations (e.g., proliferative or genomic stress) [25].

ATM (ataxia-telangiectasia mutated) is a crucial protein involved in DNA repair in response to DNA-damaging chemotherapy. ATM deficiencies may result in increased susceptibility to DNA damage and a predisposition to cancer [28]. A study by Feng et al. showed that low ATM protein expression in malignant tumor compartments contributed to the aggressive nature of breast cancer and was an independent prognostic factor associated with worse survival in patients with hormone-negative breast cancer [29]. In our study, we also observed a decrease in mRNA and protein expression for ATM in patients with ovarian cancer, which may indicate a worse prognosis for their survival.

The p53 upregulated modulator of apoptosis (PUMA), also known as Bcl-2-binding component 3 (BBC3), is a pro-apoptotic protein induced by the p53 tumor suppressor and other apoptotic stimuli [30]. As shown in studies, PUMA expression independent of p53 can also be activated, for example, by oncogenic stress [31], inhibition of kinases including FOXO [30], and altered redox [32] as well as infection [33]. Moreover, activation of both p53 and PUMA may occur following chemical-induced DNA damage and/or genotoxicity for example conventional chemotherapeutic drugs [34]. In our study, we showed an increase in mRNA and protein expression for PUMA in patients with ovarian cancer, which may be the result of oncogenic stress.

NOXA is also a BCL2 family protein that promotes apoptosis. A study by Shibue et al. showed that NOXA can selectively induce apoptosis in cancer cells [35]. Although the mechanism regarding the selectivity of apoptosis induction in cells expressing oncogenes has not yet been elucidated, it has been proposed that NOXA may require a cellular state in which cells are more sensitive to induction of apoptosis by increased expression of other pro-apoptotic factors, such as Bax [35]. Our research results do not support this suggestion because we observed a decrease in the level of NOXA gene expression in the neoplastic tissue of ovarian cancer. This suggests that the NOXA gene may also be a biomarker of worse prognosis for patient survival.

In our study, the analysis of the mRNA and protein levels showed a statistically significant decrease in c-JUN. We observed an increase in mRNA and protein levels for KRAS and c-FOS, suggesting that these oncogenes may also be biomarkers of a worse prognosis for patient survival.

As shown, members of the Fos family bind to Jun proteins and form a complex with the transcription factor AP-1, participating in the proliferation and differentiation of normal tissue, as well as oncogenic transformation and tumor progression [36]. Controversial results were obtained by Mahner et al., who showed a loss of c-FOS expression in patients with epithelial ovarian cancer, indicating that c-FOS may play a role in tumor suppression in ovarian cancer [36].

The tumor suppressor gene MEN1 encodes a protein called menin, which is related to many biological processes, including cell proliferation, migration, gene expression, and repair of DNA damage. The MEN1 gene is responsible for the occurrence of multiple proliferative changes (hyperplasia, adenomas, and carcinomas) [37]. In our study, we showed a decrease in the mRNA and protein level for MEN1 in patients with ovarian cancer compared with healthy controls. So far, the analysis of MEN1 gene expression has not been analyzed in ovarian cancer, so it is difficult to relate our results to other studies. However, our results may indicate that a decrease in MEN1 expression levels may result in increased susceptibility to DNA damage and a predisposition to cancer.

Moreover, it is believed that immune pathways are often associated with carcinogenesis. NOD1 and NOD2 are innate immune receptors that can initiate a potent immune response against pathogens. However, the role of nucleotide-binding oligomerization domain 2 (NOD2) in cancer is not well understood [38]. A study by Xu et al. showed that NOD2 maybe a biomarker for the survival of kidney cancer patients [39]. Our analysis does not confirm such a relationship because no significant changes in the transcriptional levels were observed for *NOD2* and *CHEK2.* The CHEK2 gene encodes checkpoint kinase 2 protein (CHK2), which is initially recognized as an effector kinase in the ATM–CHK2–p53 pathway responsible for a barrier preventing early carcinogenesis, including the inhibition of the cell cycle or apoptosis [40].

Moreover, we also observed an increase in the expression level of mRNA and protein for EGFR in women with ovarian cancer. The epidermal growth factor receptor (EGFR) is one of the key signaling molecules involved in the process of cell proliferation, migration, and invasion [41]. A study by Teplinsky and Muggia also found high expression of EGFR in women with ovarian tumors, which may be associated with tumor progression [42]. In another study, Cirstea et al. showed that EGFR overexpression was associated with the stage of the tumor, but their examination had limitations [43]. Similarly, Farrag et al. found no significant association between EGFR overexpression and a residual tumor or a high rate of cell proliferation [44].

## 5. Conclusions

In summary, we provided evidence that KRAS, NOXA, PUMA, c-FOS, and c-JUN could be promising biomarkers in ovarian cancer. The results of our study emphasize the usefulness of expression analysis in elucidating the genetic profiles of ovarian carcinoma. By comparing the gene expression profiles of ovarian cancer tissues with those of various other normal and malignant tissues, genes that are specific too varian cancer can be identified. These genes can be further analyzed to obtain important insight into the molecular mechanisms involved in ovarian carcinogenesis. Moreover, these genes can also be tested as potential markers of ovarian cancer and may contribute to the diagnosis and/or treatment of ovarian cancer.

Therefore, there are some limitations to our study. First of all, the study group was, in most cases, diagnosed with advanced ovarian cancer, so it is worth extending the study group to early cases of ovarian cancer development. This will make it possible to compare the expression level of the analyzed genes between women with advanced ovarian cancer and patients in the early stages of the disease. Moreover, it will be interesting to investigate whether these reported markers in ovarian cancer tissue show a similar effect in serum samples, potentially providing a better, mildly invasive patient approach. This could provide valuable information for ovarian cancer patients with regard to diagnosis, treatment, and prognosis.

## Figures and Tables

**Table 1:** Sequences of primers used for real-time PCR.

Gene	Forward (5′-3′)	Reverse (5′-3′)	Reference
ATM	GGTATAGAAAAGCACCAGTCCAGTATTG	CGTGAACACCGGACAAGAGTTT	[12]
BRCA1	GCATGCTGAAACTTCTCAACCA	GTGTCAAGCTGAAAAGCACAAATGA	[12]
BRCA2	AGACTGTACTTCAGGGCCGTACA	GGCTGAGACAGGTGTGGAAACA	[12]
KRAS	TCTTGCCTCCCTACCTTCCACAT	CTGTCAGATTCTCTTGAGCCCTG	[13]
c-JUN	ACCTTCAACACCCCAGCCATG	GGCCATCTCTTGCTCGAAGTC	[14]
c-FOS	GCCTCGTTCCTCCAGTCCGA	TGCGATGGAAAGGCCAGCCC	[14]
NOXA	GCTGGAAGTC GAGTGTGCTA	CCTGAGCAGAAGAGTTTGGA	[15]
PUMA	GCCAGATTTGTGAGACAAGAGG	CAGGCACCTAATTGGGCTC	[16]
MEN1	CTTCCATTGACCTGCACACC	CAGCCAGGTACATGTAGGG	[17]
NOD2	ACCTTTGATGGCTTTGACG	CACCTTGCGGGCATTCTT	[18]
CHEK2	TCAGCAAGAGAGGCAGACCC	ACAGCTCTCCCCCTTCCATC	[12]
EGFR	TGATAGACGCAGATAGTCGCC	TCAGGGCACGGTAGAAGTTG	[13]
GAPDH	GCAAATTCCATGGCACCGT	TCGCCCCACTTGATTTTGG	[12]
β-ACTIN	GCCAGAGCGGGAGTGGTGAA	GGCTTGGGCTCAGGGTCATT	[14]

ATM-ATM Serine/Threonine Kinase, BRCA1-BRCA1 DNA Repair Associated, BRCA2-BRCA2 DNA Repair Associated, KRAS-Kirsten rat sarcoma viral oncogene homolog, c-JUN-Jun proto-oncogene (AP-1 transcription factor subunit), c-FOS-Fos proto-oncogene (AP-1 transcription factor subunit), NOXA-phorbol-12-myristate-13-acetate-induced protein 1, PUMA-p53 upregulated modulator of apoptosis, MEN1-menin 1, NOD2-nucleotide binding oligomerization domain containing, CHEK2-checkpoint kinase 2, EGFR-epidermal growth factor receptor, GAPDH-glyceraldehyde-3-phosphate dehydrogenase.

**Table 2:** Comparison of selected clinical and biochemical parameters between women with ovarian cancer and control groups.

Parameter	Group	Mean ± SEM	Median	95% CI	*p*
Leukocytes 10^9^/L	OC	8.24 ± 3.48	7.32	7.53–8.94	0.006
	Control	6.93 ± 2.61	6.57	6.41–7.45	
Erythrocytes 10^12^/L	OC	4.28 ± 0.58	4.32	4.16–4.40	0.148
	Control	4.39 ± 0.53	4.41	4.28–4.49	
Platelets 10^9^/L	OC	328.87 ± 158.44	287.00	296.76–360.96	0.014
	Control	266.48 ± 64.87	262.50	253.47–279.48	
Hemoglobin g/dL	OC	7.46 ± 1.01	7.50	7.26–7.67	0.035
	Control	7.71 ± 0.91	7.80	7.52–7.89	
Hematocrit	OC	0.37 ± 0.36	0.37	0.36–0.38	0.059
	Control	0.46 ± 0.83	0.38	0.30–0.63	
Glucose mg/dL	OC	96.12 ± 18.28	92.00	92.41–99.82	<0.001
	Control	88.81 ± 14.96	84.85	71.22–91.81	
Sodium mmol/L	OC	139.50 ± 2.89	138.91	138.91–140.09	0.035
	Control	139.28 ± 2.60	139.00	138.75–139.80	
Potassium mmol/L	OC	4.37 ± 0.43	4.37	4.29–4.46	0.417
	Control	4.25 ± 0.31	4.21	4.19–4.32	
Creatinine mg/dL	OC	0.84 ± 0.49	0.73	0.73–0.95	0.631
	Control	0.82 ± 0.24	0.76	0.70–0.94	
eGFRmL/min/1.73 m^2^	OC	85.53 ± 33.53	87.21	77.22–93.84	0.289
	Control	94.97 ± 22.65	96.95	82.91–107.04	
Total protein g/dL	OC	6.93 ± 0.81	6.95	6.74–7.12	0.314
	Control	7.07 ± 0.40	7.05	6.88–7.28	
Uric acid mg/dL	OC	5.08 ± 1.77	4.80	4.67–5.49	0.714
	Control	5.14 ± 1.51	5.10	4.36–5.92	
Urea mg/dL	OC	33.30 ± 18.89	28.70	28.22–37.65	0.276
	Control	32.42 ± 10.38	30.00	27.09–37.77	
D-dimer ng/mL	OC	3329.63 ± 2124.62	1831.00	2459.53–4199.53	<0.001
	Control	789.69 ± 423.54	464.50	397.20–1182.18	
Fibrinogen g/L	OC	7.55 ± 6.34	3.62	0.25–14.84	<0.001
	Control	2.91 ± 0.75	2.89	2.61–3.20	
INR	OC	1.17 ± 0.23	1.12	1.12–1.22	0.073
	Control	1.09 ± 0.05	1.11	1.07–1.12	
PTT	OC	12.92 ± 2.52	12.40	12.36–13.47	0.058
	Control	12.08 ± 0.65	12.20	11.81–12.35	
APTT	OC	30.00 ± 3.82	30.20	29.17–30.84	0.955
	Control	30.31 ± 3.72	30.45	28.74–31.88	
Systolic pressure mmHg	OC	123.07 ± 13.17	125.00	120.39–125.76	0.165
	Control	120.81 ± 14.98	120.00	117.81–123.82	
Diastolic pressure mmHg	OC	78.94 ± 14.59	80.00	75.98–81.89	0.719
	Control	78.32 ± 8.45	80.00	76.62–80.01	
CA-125 U/mL	OC	773.51–454.22	290.00	501.27–1045.74	<0.001
	Control	128.29 ± 100.43	20.81	8.49–266.64	
HE4 pmol/L	OC	1708.42 ± 1204.42	363.45	129.20–3967.74	0.009
	Control	83.47 ± 30.34	73.66	8.12–158.83	

INR—international normalized ratio, PTT—prothrombin time, APTT—activated partial thromboplastin time, OC—women with ovarian cancer, eGFR—glomerular filtration rate.

**Table 3:** Summary of mRNA expression analysis of selected genes in patients with ovarian cancer in comparison to the control group.

Gene	Patients with Ovarian Cancer	*p*-Value
ATM	75.45 ± 5.56	0.056
BRCA1	107.69 ± 9.42	0.231
BRCA2	120.00 ± 12.24	0.084
CHEK2	97.50 ± 8.45	0.142
KRAS	195.55 ± 18.32	**0.012**
NOXA	51.96 ± 7.34	**0.024**
PUMA	184.97 ± 18.42	**0.026**
c-FOS	228.57 ± 21.44	**0.008**
EGFR	186.66 ± 17.54	**0.006**
NOD2	84.61 ± 9.32	0.164
MEN1	69.01 ± 8.64	0.118
c-JUN	34.14 ± 6.42	**0.001**

Values are presented as ratios against mRNA GAPDH/β-ACTIN expression. The control group was defined as 100%. Data are presented as mean% ± SD, *p* < 0.05 compared with the control group. ATM-ATM Serine/Threonine Kinase, BRCA1-BRCA1 DNA Repair Associated, BRCA2-BRCA2 DNA Repair Associated, KRAS-Kirsten rat sarcoma viral oncogene homolog, c-JUN-Jun proto-oncogene (AP-1 transcription factor subunit), c-FOS-Fos proto-oncogene (AP-1 transcription factor subunit), NOXA-phorbol-12-myristate-13-acetate-induced protein 1, PUMA-p53 upregulated modulator of apoptosis, MEN1-menin 1, NOD2-nucleotide binding oligomerization domain containing, CHEK2-checkpoint kinase 2, EGFR-epidermal growth factor receptor.

**Table 4:** Analysis of the protein level for ATM, BRCA1, BRCA2, KRAS, c-JUN, c-FOS, NOXA, PUMA, MEN1, NOD2, CHEK2, and EGFR in the tissue homogenates in patients with ovarian cancer in comparison to the control group.

Gene	Patients with Ovarian Cancer	*p*-Value
ATM	89.64 ± 9.32	0.086
BRCA1	98.67 ± 6.41	0.224
BRCA2	92.28 ± 9.45	0.196
CHEK2	88.26 ± 11.24	0.252
KRAS	141.11 ± 8.65	**0.044**
NOXA	60.62 ± 6.56	**0.042**
PUMA	115.73 ± 9.66	0.052
c-FOS	145.52 ± 10.48	**0.032**
EGFR	178.89 ± 7.32	**0.046**
NOD2	86.08 ± 10.11	0.262
MEN1	82.35 ± 7.44	0.275
c-JUN	54.43 ± 9.82	**0.024**

The control group was defined as 100%. Data are presented as mean% ± SD, *p* < 0.05 compared with the control group. ATM-ATM Serine/Threonine Kinase, BRCA1-BRCA1 DNA Repair Associated, BRCA2-BRCA2 DNA Repair Associated, KRAS-Kirsten rat sarcoma viral oncogene homolog, c-JUN-Jun proto-oncogene (AP-1 transcription factor subunit), c-FOS-Fos proto-oncogene (AP-1 transcription factor subunit), NOXA-phorbol-12-myristate-13-acetate-induced protein 1, PUMA-p53 upregulated modulator of apoptosis, MEN1-menin 1, NOD2-nucleotide binding oligomerization domain containing, CHEK2-checkpoint kinase 2, EGFR-epidermal growth factor receptor.

## Data Availability

Not applicable.
